# Supplementary material for: Microbial biofilms as living photoconductors due to ultrafast electron transfer in cytochrome OmcS nanowires
Source: Nat Commun. 2022 Sep 7;13:5150. doi: 10.1038/s41467-022-32659-5 (PMC9452534; doi:10.1038/s41467-022-32659-5)
Supplement: Supplementary file 1 — Supplementary Information [file 41467_2022_32659_MOESM1_ESM.pdf]

Supplementary Information

Microbial biofilms as living photoconductors due to ultrafast electron transfer in cytochrome OmcS nanowires

Jens Neu[1,2,5*], Catharine C. Shipps[1,2,5], Matthew J. Guberman-Pfeffer[1,2], Cong Shen[1,2], Vishok Srikanth[1,2], Jacob A. Spies[3], Nathan D. Kirchhofer[4], Sibel Ebru Yalcin[1,2], Gary W. Brudvig[3], Victor S. Batista[3], and Nikhil S. Malvankar[1,2*]

This file contains

Supplementary Figures 1-11

Supplementary Tables 1-2

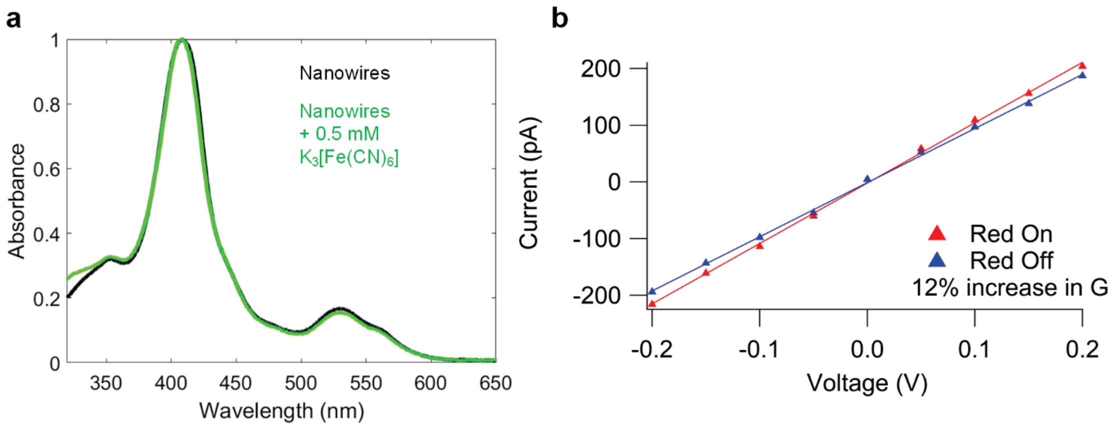

SI Fig. 1. Nanowires are fully oxidized in air and chemically-reduced nanowires lack photoconductivity. a, The UV-Vis spectra of nanowires are identical without (black) and with (green) potassium ferricyanide confirming that the nanowires are fully oxidized in air. **b,** Chemically reduced nanowires show very small (12%) increase in conductance (G) upon photoexcitation. Red curve – laser on. Blue curve – laser off.

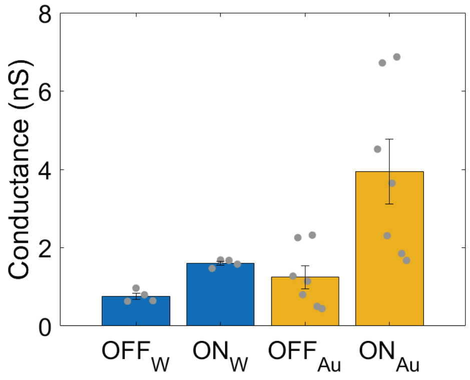

SI Figure 2. Nanowires show photoconductivity irrespective of electrode materials. Upon photoexcitation, nanowire conductance increased 110% on tungsten electrodes with 10 µm spacing (blue) and 230% on gold electrodes with 5 µm spacing (yellow). Individual data points are shown as grey dots. Values represent mean ± standard error of the mean (S.E.M) (n=4 independent devices for tungsten and 7 devices for gold).

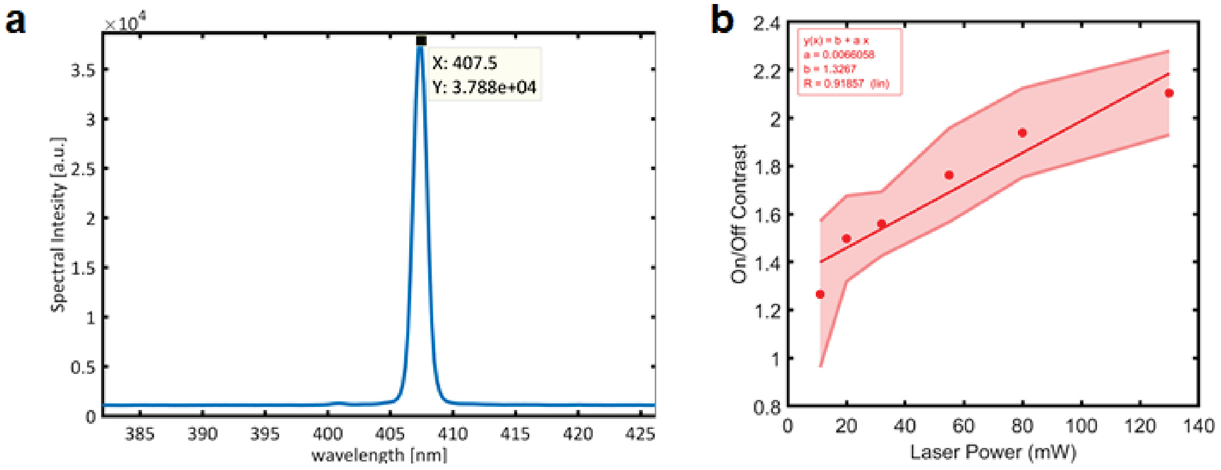

SI Fig. 3. Nanowire conductance upon laser on/off increases with laser power. a, Laser characterization for network measurements. **b,** Ratio of current for nanowires with laser on and laser off under 200 mV bias with a linear fit shown by a solid line. Values represent mean ± S.D. (shaded region) for three independent samples.

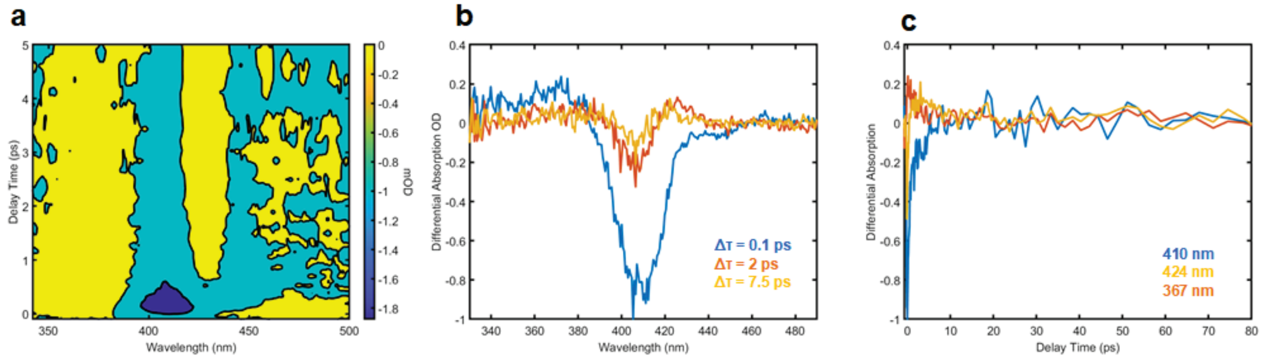

Supplementary Figure 4. Ultrafast (<100 fs) charge transfer between hemes in nanowires revealed by fs-TA with a pump beam at λ=530 nm. a, 2-D map of differential absorption over the probed wavelengths and delay time. **b**, Change in differential absorption over wavelength at different delay times as stated in legend. **c**, Change in differential absorption over delay time at key wavelengths.

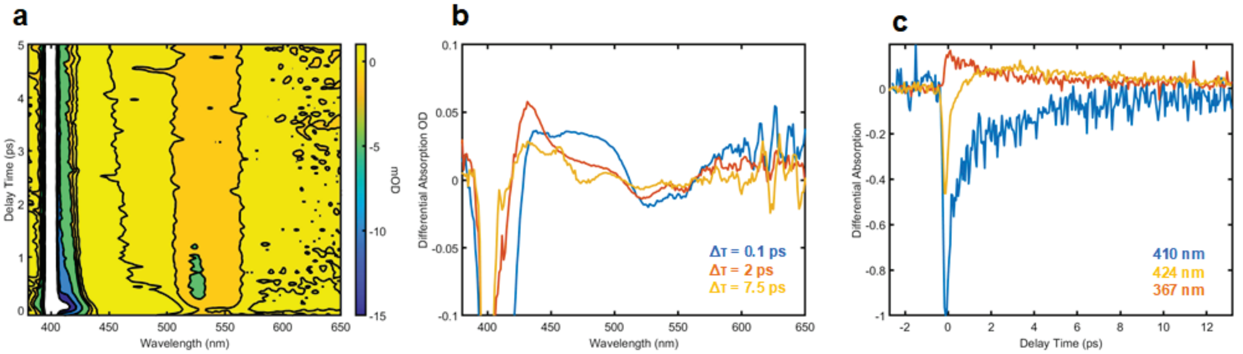

Supplementary Figure 5. Ultrafast (<100 fs) charge transfer between hemes in nanowires revealed by fs-TA with a pump beam (λ=400 nm). a, 2-D map of differential absorption over the probed wavelengths and delay time. **b**, Change in differential absorption over wavelength at different delay times as stated in legend. **c**, Change in differential absorption over delay time at key wavelengths.

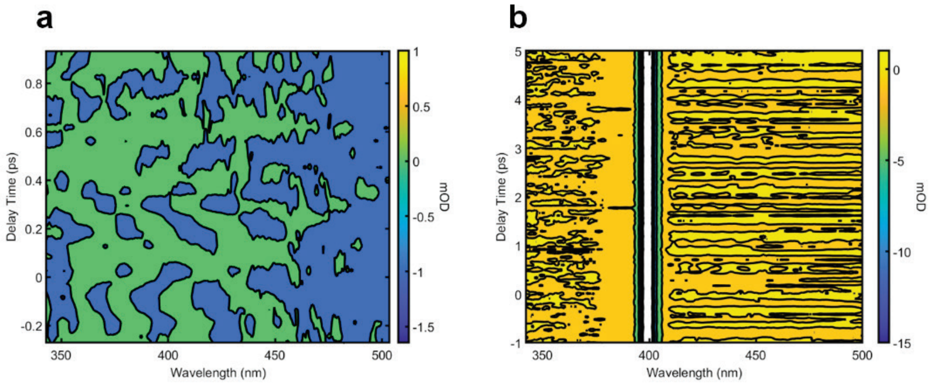

Supplementary Figure 6. Lack of charge transfer without nanowires. a, 2-D map of differential absorption over the probed wavelengths and delay time shows lack of charge transfer in the substrate alone without nanowires and **b,** lack of charge transfer in the buffer alone without nanowires.

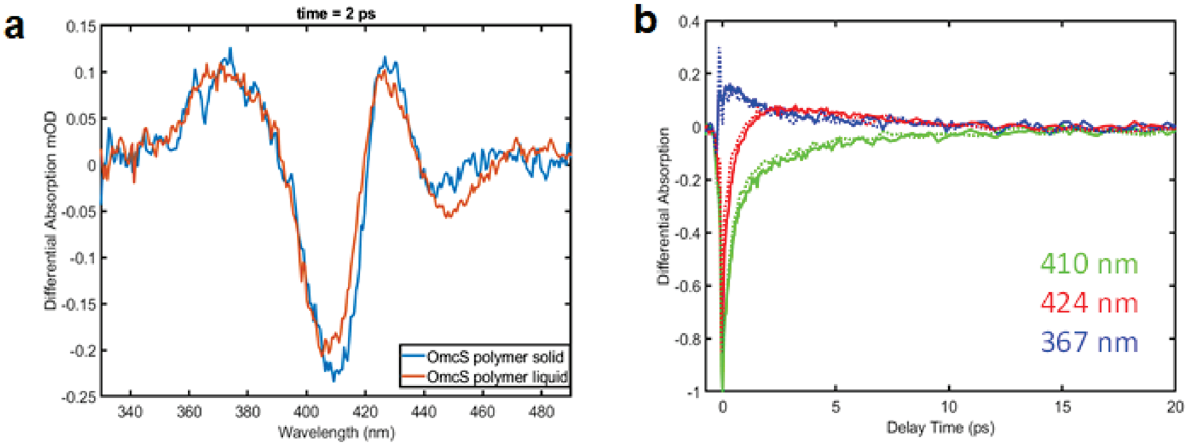

Supplementary Figure 7. Solid state and liquid measurements give similar dynamics. a, Change in differential absorption over wavelength 2 ps after photoexcitation. **b,** Change in differential absorption over delay time at key wavelengths; solid line shows solid state and dashed lines are liquid state.

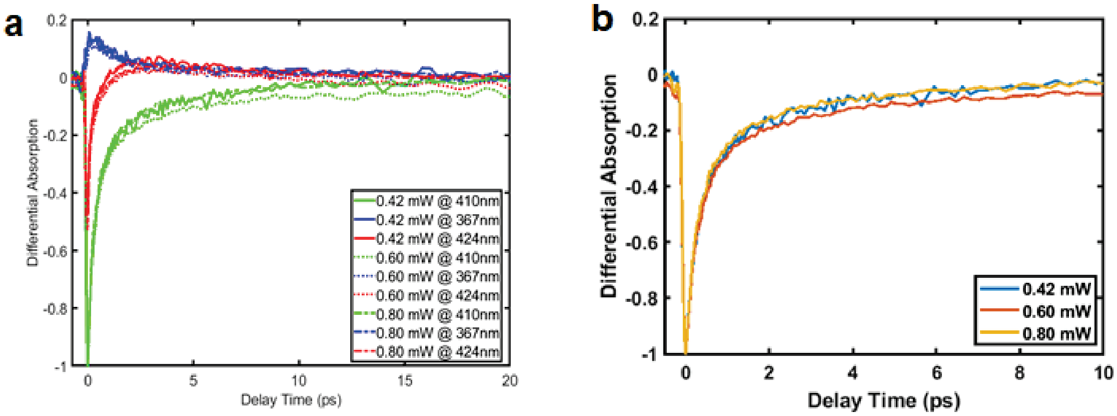

Supplementary Figure 8. Transient absorption dynamics are independent of laser power. a, Normalized change in differential absorption over delay time. **b,** Representative Normalized dynamics for 410 nm wavelength.

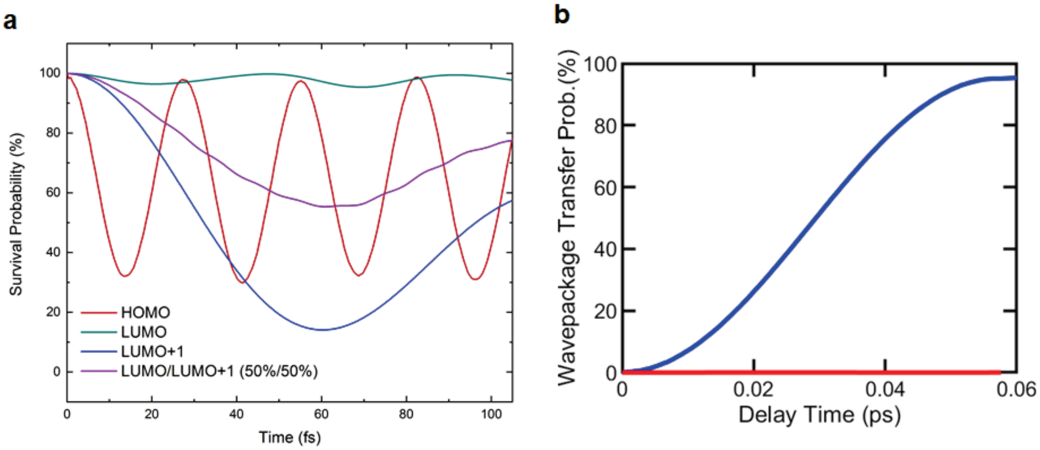

Supplementary Figure 9. a, Survival probability of an electron (likelihood to remain on the heme) starting in each initial state on a slip stacked pair of hemes. Electron transfer is likely (< 60% survival probability) for most energy levels. **b,** Wavefunction transfer probability for parallel-stacked (blue) vs. perpendicular stacked hemes (red)

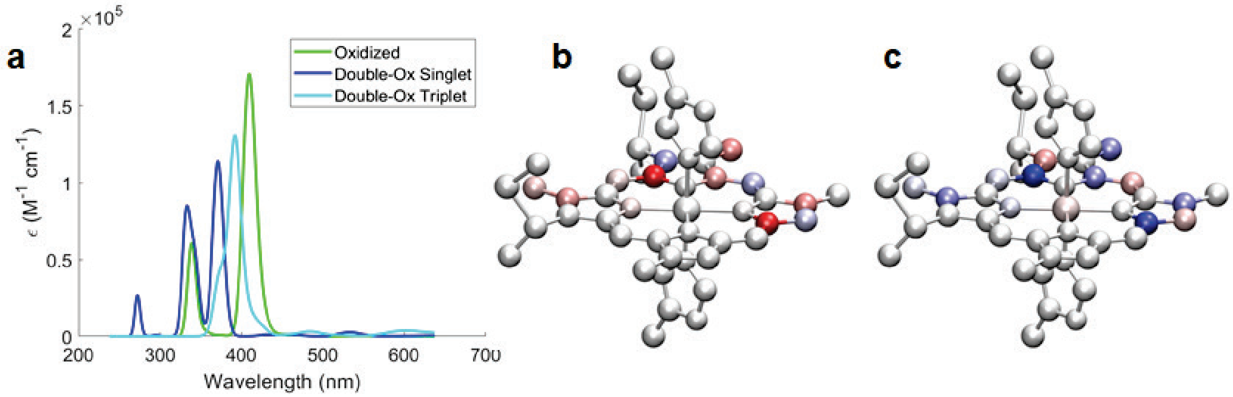

Supplementary Figure 10. Atomic spin populations for doubly oxidized species. a, Simulated UV-Vis spectra for doubly oxidized species in singlet and triplet state compared to the ground state. Representation of the change in Mulliken atomic spin populations upon conversion of the singly oxidized heme in the doublet spin state into either the open-shell singlet (**b**) or triplet (**c**) doubly oxidized species. Coloration from blue to red indicate positive to negative changes in spin populations along a symmetric colour range between ±0.13. Note that there is hardly any change in the spin population on the Fe centre as a result of the second oxidation.

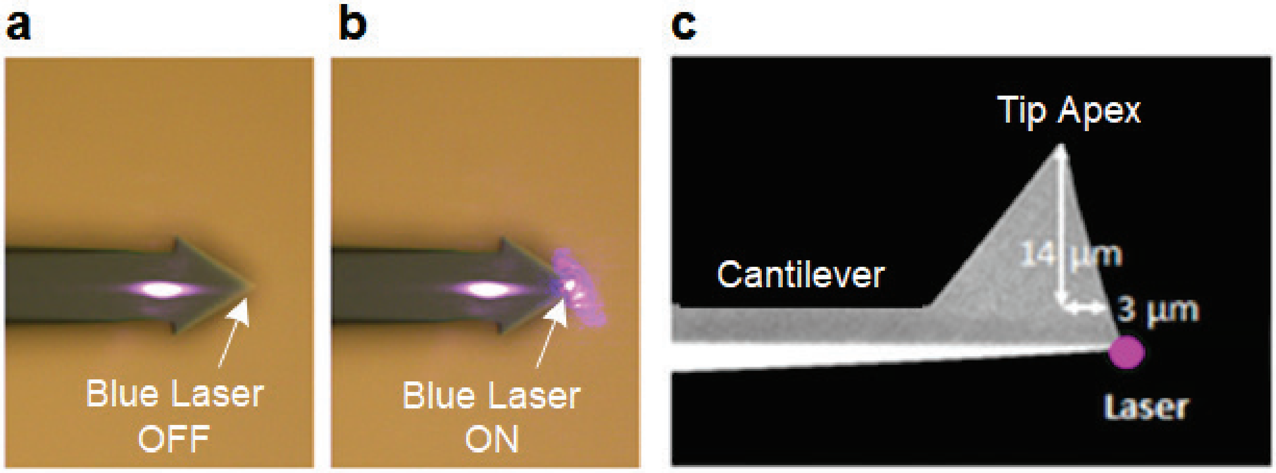

Supplementary Figure 11. Laser alignment for Pc-AFM. a, Top view of the cantilever no blue laser applied (Laser OFF). **b**, Blue laser aligned to cantilever (Laser ON). **c**, Side view of cantilever with schematic showing the laser aligned to the cantilever (purple dot) and the angle between the laser alignment and tip. Image modified from manufacturer's website. The laser power that reaches nanowires will be much lower than the applied power because the laser is directly focused on the cantilever not the tip apex below which the nanowires are probed.

Supplementary Table 1. Summary of Mulliken atomic spin populations upon second oxidation of the heme cofactor

	Singly Oxidized (Doublet)	Doubly Oxidized (Open Singlet)	Doubly Oxidized (Triplet)
Fe	1.038	1.037	1.022
Ligands[a]	-0.039	-1.036	0.980
Total	1.000	0.001	2.002

[a]The ligands to the Fe centre include the tetrapyrrolic macrocycle with all substituents (except the propionic acid groups) and 4-methylimidazole axial ligands

Supplementary Table 2. The number of conductance values used to calculate the average conductance of each nanowire with the laser ON and the laser OFF under cp-AFM.

NW	N used to calc. AVG.		NW	N used to calc. AVG.	
	ON	OFF		ON	OFF
1	40	10	9	10	30
2	80	40	10	10	10
3	100	20	11	10	10
4	20	40	12	120	70
5	40	60	13	20	120
6	40	20	14	20	40
7	80	80	15	20	40
8	20	40			
