# Supplementary material for: Microbial biofilms as living photoconductors due to ultrafast electron transfer in cytochrome OmcS nanowires
Source: Nat Commun. 2022 Sep 7;13:5150. doi: 10.1038/s41467-022-32659-5 (PMC9452534; doi:10.1038/s41467-022-32659-5)
Supplement: Supplementary file 3 — Description of Additional Supplementary Files [file 41467_2022_32659_MOESM3_ESM.docx]

**Description of Additional Supplementary Files**

**File Name:** Supplementary Data 1

**Description:** Structures used for both optical spectra simulations and quantum dynamics simulations are provided as Supplementary Data 1.
